# The Dialogical Jung: Otherness within the Self

**DOI:** 10.3390/bs3040634

**Published:** 2013-11-21

**Authors:** William E. Smythe

**Affiliations:** Psychology Department, University of Regina, 3737 Wascana Parkway, Regina S4S 0A2, SK, Canada; E-Mail: william.smythe@uregina.ca; Tel.: +306-585-4219; Fax: +306-585-4772

**Keywords:** analytical psychology, dialogical self, archetypes, otherness

## Abstract

This paper explores dialogical currents in Jung’s analytical psychology, with reference to contemporary theories of the dialogical self. The dialogical self is a notion that has gained increasing currency in psychology since the 1990s, in response to the limitations of traditional notions of the self, based on monological, encapsulated consciousness. Modern dialogical self theory construes the self as irrevocably embedded in a matrix of real and imagined dialogues with others. The theme of dialogical otherness within the self is also taken up in Jung’s analytical psychology, both in the practice of active imagination and psychotherapy and in the theory of archetypes, and a dialogical approach to inquiry is evident in Jung’s work from the outset. The implications of a dialogical re-conceptualization of analytical psychology and of analytical psychology for dialogical theory are considered in detail.

## 1. Introduction

In a recent essay Charles Taylor identified the problem of understanding the other as “the great challenge of this century both for politics and social science” ([1], p.24). What analytical psychology can uniquely contribute with respect to this challenge is to give an account of the other within the self. This paper takes up the theme of otherness in Jung’s analytical psychology from the perspective of contemporary theories of the dialogical self. I begin by reviewing current notions of the dialogical self, focusing on the seminal theory of Hubert Hermans and colleagues. Next, I examine some dialogical currents in Jungian thought from early childhood experiences to the pattern of dialogical engagement portrayed in Jung’s *The Red Book* [2], to his account of dialogical psychotherapy, to the apotheosis of Jung’s understanding of dialogical otherness in the archetype of Self. Critical to the discussion is the notion of *background understanding* and the different ways it is understood in contemporary dialogical theory *versus* Jung’s archetypal theory, and the distinction between dialogue and dialectics. I conclude that Jung’s approach to dialogical thinking can both inform and be informed by current developments in dialogical self theory.

## 2. The Dialogical Self

The dialogical self has attracted a good deal of attention and discussion since the notion was first seriously taken up in psychology in the early 1990s. The proliferation of recent academic volumes, articles, special issues and conferences devoted to the topic is noteworthy, especially in light of the fact that, as Stam [3] has pointed out, the basic idea of a dialogical self is still far from clear. In some respects the dialogical self is a thoroughly familiar aspect of everyday experience. We all find ourselves inescapably embedded in a world of others, whose actual and virtual presence continually shapes our self-understanding in ways we cannot fail to notice. Even our most personal and private reflections are invariably saturated with the voices and perspectives of others urging us on, cajoling, criticizing, praising and pleading with us, seemingly at every turn. Yet, as is often the case, it takes theory some time to catch up with common experience. Much of our theoretical and conceptual discourse about the self still remains in the grip of a centuries-old epistemological tradition that goes all the way back to Descartes and Locke and was fueled by 19th Century notions of privacy and self-scrutiny [4,5,6]. This is the traditional, monological picture of disengaged, first-person, “radically reflective” subjectivity that still underlies much of what we say *about* the self as an abstract concept. However, our day-to-day experience in actually negotiating our sense of self in the world obviously suggests a different picture.

### 2.1. The Hermans Formulation

The formulation of the dialogical self that has had the most influence in psychology is that of Hubert Hermans and colleagues [7,8,9,10,11], who define the *dialogical self* as a dynamic multiplicity of voiced positions in an extended dialogical landscape of mind that includes actual others in the social world and imagined others that are intimately intertwined with them. Self, on this conception, is both multivoiced and dialogical. In expressing and reflecting on one’s self, one can be said to occupy any number of distinct positions in dialogical space and to give voice to them in unique ways. In this formulation it is critical to distinguish between *voices* and *positions*, where *voice* refers to “the motivated, emotional, and agentic starting point of a message that is addressed to any other person or to another part of the self” and *position* refers to “the place where a voice is located in an imaginal [dialogical] space” ([12], p. 380). This merging of voices with positions in dialogical space is said to be a function of the agency of the “I”, which “has the capacity imaginatively to endow each position with a voice” ([7], p. 148). The composite *I-positions* that result from this imaginative endowment are said to reflect both continuity and discontinuity in the dialogical self [9]. Discontinuity derives from the diverse and often contradictory character of the positions that the self takes up, whereas continuity is said to be provided by the “I” itself, insofar as “it is *one and the same I* who is doing this” ([8], p. 139). The essence of the dialogical self in any case is to be found in the continual movement among positions in dialogical space—a process called *positioning*, which is undertaken not only in relation to others but also in relation to oneself [8].

Although Hermans and colleagues, in a recent publication, have dismissed the notion of a singular self as a mere “trick of grammar” ([12], p. 380), they nonetheless invest the “I” with substantial agentic powers to endow dialogical positions with voice. The dialogical self, on this view, is identified with its possibilities for agentic movement in dialogical space, in terms of which it voices its various possibilities. One might well question whether the kind of radical dialogical freedom implied by this view is always a good thing; could it not also tend to foster confusion, disorientation and indecisiveness in one’s sense of self?

### 2.2. The Dialogical Background

In any case, to focus in this way on the agentic possibilities of the dialogical self is to overlook its grounding in pre-intentional and inarticulate dialogical processes. This is a common theme both in early formulations of the dialogical self, prior to the Hermans work [6], and also in recent critiques [13,14,15,16]. Burkitt, for example, questions the status of Hermans’s agentic, dialogical “I” as “something that originally exists apart from voice with the ability to move at will between positions and voices, seemingly animated by its own agency”; this, he points out, overlooks “the sense of ‘otherness’ within the self: that from the earliest years our sense of self is intertwined with the voices of others, often in unwanted, unplanned, unwelcome, and surprising ways” ([14], p. 306). That is, long before we can exercise the capacity for intentional positioning in dialogical space, in the way that Hermans and colleagues envision, our dialogical selves are already formed by the pre-intentional and inarticulate matrix of our relations with others. The dialogical self does not just range freely over positions and voices in dialogical space, then, it is also fundamentally *constituted* by them. What the Hermans *et al.* conception of the agentic dialogical self tends to overlook is the ways in which dialogue is inherently *constitutive* of self.

Charles Taylor [6] understands the dialogical constitution of self as a form of *background understanding*. Background understanding, or *pre-understanding*, is a notion that has played a fundamental role in modern hermeneutic philosophy and in philosophical discussions of language and meaning generally [17,18,19,20]. It refers to the tacit, inarticulate, taken-for-granted contexts of human meaning that are grounded in our embodied capacities, dispositions, shared practices and forms of life, which constitute a fundamental condition of intelligibility of meaningful human activities and expressions.

Our articulate and intentional construals of self, then, inevitably presuppose and are grounded in a tacit background of situated and embodied practices that cannot in the nature of things be made fully explicit. These practices are, from our earliest moments of experience, grounded in our shared life with others and in forms of dialogical activity that possess what Taylor [6] called a “common rhythm” long before they have any articulate content. The dialogical structures that are deeply buried in these common rhythms of communal life remain generally inaccessible to conscious awareness; as Martsin has more recently observed, “we cannot and usually do not need to talk about this invisible and taken-for-granted background, yet it constantly regulates our way of being as our new encounters with the world are made sense of in relation to it” ([16], p. 439). It is only when there is a significant disruption in the flow of our coordinated activities with others that the full range of dialogical possibilities of self begins to come to consciousness. As Martsin went on to point out, it is in these “moments of rupture” that “our united and backgrounded sense of being becomes foregrounded and multiple...When we look at these moments of rupture we see no unified sense of identity, bur multiple situation-bound ways of defining our fuzzy sense of being” ([16], p. 441). Moreover, inasmuch as we cannot fully extricate ourselves from the concrete contexts of our background dialogical understanding, the sense of otherness that in this way comes into the self is inevitably partial, fragmentary and unknowable.

Barresi [21] further analyzed the epistemological limits of the dialogical self in terms of an important distinction between *first-person* (actor’s) and *third-person* (observer’s) perspectives on dialogical self-understanding. Whereas our own current first-person activities and third-person information about the activities of others with whom we are currently involved are both directly witnessed, the first-person perspective of another and a third-person perspective toward oneself can only be imagined. We resort to imagination in these instances in an attempt to fill gaps that cannot be filled by direct experience and, as Barresi noted, “imagination can never fully achieve that job” ([21], p. 246). The reason is that we can never, even in our most vivid acts of imagination, actually inhabit the embodied, first-person experience of another nor entertain a fully objective, third-person view of ourselves. 

The other within the dialogical self cannot be an object of explicit, discursive knowledge, then. In its more tacit form it lies buried in our background understanding of the social world; in its more active or intentional form, it is occluded in the fundamental asymmetry between first- and third-person knowledge. It is not something that can be objectively known but only imaginally projected; it is fundamentally an “as if” [22].

### 2.3. Dialogue and Dialectics

Critical to an understanding of dialogical “otherness” within the self is the distinction between dialogue, or “dialogic”, and the closely related notion of *dialectic*. The origins of dialectic go back in the Western tradition to the ancient Greek philosophers, where it finds its most complete expression in the Socratic dialogues of Plato. In this context, dialectic was understood as a method of rational argument based on dialogue between two or more individuals who hold contrary positions and proceeds by identifying contradictions and inconsistencies in one’s assumptions along the way toward discovering truth. Beginning with Hegel, dialectic subsequently came to be understood as a fundamental aspect of thought and reality itself, not just of discourse. In particular, dialectic is evident in the dynamic interplay between contradictory or oppositional parts of any living process and their subsequent resolution or integration into a new synthesis. Dialectical materialism derives from a Marxist recasting of the Hegelian dialectic in terms of concrete historical and economic processes [23,24]. Common to the myriad forms of dialectic is the notion of dynamic interaction among pairs of opposites and the emergence some form of resolving synthesis.

Dialogical thought, in contrast to dialectics, involves constituents that coexist but are not generally resolved or synthesized into a new whole. Writing of the polyphonic novel, for example, Bakhtin asserted that each such work “presents an opposition, which is never cancelled out dialectically, of many consciousnesses, and they do not merge in the unity of an evolving spirit” ([25], p. 26). In Bakhtin’s dialogism, all language and thought is seen to be in response to past utterances and in anticipation of future ones, such that any given expression is always thoroughly saturated and enmeshed with the voices of others, which is the essence of what he termed *polyphony*. These mutually entangled voices and perspectives do not finally converge into a coherent overall picture but, rather, coexist in an unresolved plurality of “unmerged voices and consciousnesses” ([25], p. 6). Whereas dialectical thinking is based on tension and resolution among pairs of opposites, dialogical thinking is based, not merely on *opposition*, but on *otherness*; and, while dialectical opposites are to be resolved, integrated or transcended, dialogical others can coexist in polyphonic plurality of voices and perspectives. As Hermans [26] has shown, characters in the dialogical self frequently arrange themselves in pairs of opposites but dialogical self theory also makes room for an otherness within the self that cannot be resolved or synthesized dialectically.

## 3. Dialogical Elements in Analytical Psychology

The theme of dialogical otherness within the self is also taken up in Jung’s analytical psychology, which predates the modern dialogical self tradition by several decades but can nonetheless inform and be informed by it. Papadopoulos [27,28] has extensively documented the theme of “otherness” in Jung’s life and work, making a compelling case that this was an essential preoccupation of Jung’s throughout his long and productive career. The contributors to a recent volume edited by Jones and Morioka [29] focus more specifically on *dialogical* aspects of Jung’s approach to otherness within the self, which is the focus of the present paper as well. The aim is not to attempt to assimilate or “domesticate” analytical psychology to the categories of dialogical self theory but, rather, to extend the reach of both traditions through mutual engagement. Although the notion of dialogue does not receive systematic treatment in the corpus of Jung’s theorizing, it is evident that he was in many respects a dialogical thinker from the outset.

### 3.1. Childhood Experiences

During his early childhood years, for example, Jung often played an imaginary game with a favorite stone in the garden of his family home, which, as he later described it,

went something like this: “I am sitting on top of this stone and it is underneath.” But the stone also could say “I” and think: “I am lying here on this slope and he is sitting on top of me.” The question then arose: “Am I the one who is sitting on the stone, or am I the stone on which *he* is sitting?” This question always perplexed me, and I would stand up, wondering who was what now.

([30], p. 20)

Jung’s feeling of perplexity and uncertainty in playing this simple game was also accompanied by a distinct sense of curiosity and fascination. This was among a series of childhood games Jung invented, in which he imagined himself in a “secret relationship” with an object; in the case of the stone, the relationship was a fluid and reversible one. Plainly, the notion of dialogical positioning of self was not unknown to Jung even as a young child.

Jung’s experience of a dialogical other within himself developed further when, by the age of 12, wrote Jung, “it occurred to me that I was actually two different persons. One of them was the schoolboy who…was far from sure of himself; the other was important, a high authority, a man not to be trifled with…an old man who lived in the eighteenth century” ([30], pp. 33–34). This marked the first appearance of what Jung came to call his “No. 1 and No. 2 personalities”, a motif that would run through the rest of his life. Personality No. 1 represented his ordinary sense of self, the son of his parents who attended school along with other children, whereas No. 2 was a mysterious “other” whose character was archaic and close to nature but remote from the world of everyday experience. Nonetheless, he came to recognize No. 2 as a bona fide aspect of himself and the dynamic interplay between No. 1 and No. 2 as something that takes place in others as well. Jung wrote:

The play and counterplay between personalities No. 1 and No. 2, which has run through my whole life, has nothing to do with a “split” or dissociation in the ordinary medical sense. On the contrary, it is played out in every individual. In my life No. 2 has been of prime importance, and I have always tried to make room for anything that wanted to come to me from within.

([30], p. 45)

### 3.2. Dialogue in The Red Book

In 1913, Jung’s willingness to open up to the world of No. 2 was put to a decisive test. Following his break with Freud, Jung went through a period of profound psychological disorientation marked by a series of disturbing dreams and visions. In an effort to come to grips with this visionary material, Jung set out over a series of evenings to “drop” down into it and subsequently record what he experienced. The initial record of these extraordinary experiences was in a series of notebooks, as yet unpublished, called the *Black Books*. A reworked and highly stylized version of some of this material, including a calligraphic transcription of the text and abundant pictorial illustrations, constitutes what became known as *The Red Book*. Jung felt that this elaborate format was the best way to do justice to the richness of his original experiences. This work was finally published in 2009 in a full sized facsimile edition that includes a lengthy historical introduction and an extensive scholarly apparatus by Editor, Sonu Shamdasani [2].

At the core of the textual content of *The Red Book* is a narrative sequence of encounters with a host of imaginary figures, some loosely based and Biblical and mythological sources, others being more or less free creations. An example of the latter is a figure called “The Red One”, who appears at the outset of *Liber Secundus*, the second part of *The Red Book*. Jung’s confrontation with The Red One, like his encounters with other figures in the work, is thoroughly dialogical. Jung’s “I”, as protagonist and narrator, does not merely listen to and record what the Red One tells him but actively questions, disputes with, challenges and even at times contradicts him. The first question he asks is “who are you?” ([2], p. 259), which The Red One refuses to answer in a straightforward way, although he is aware that he has been taken to be the devil. In the conversation that ensues, The Red One states that he finds Jung’s protagonist to be “an unbelievably ponderous and serious person” ([2], p. 259), whom he encourages to abandon seriousness and instead to “dance through life” ([2], p. 260). Jung’s “I” meanwhile defends his seriousness but acknowledges that, although he knows how to dance, he has not yet found the way to joyful expression before God that dancing could evoke; to this, The Red One replies: “brother, I am joy” ([2], p. 260). Following the conversation, while Jung’s “I” does not abandon his initial assessment of The Red One as the devil, he is now prepared to assimilate him as “my devil…the joy of the serious person” ([2], p. 260).

In a subsequent episode, Jung’s “I” meets Ammonius, the Anchorite, who is in many respects an opposite character to The Red One. Upon their initial meeting he finds Ammonius sitting in a desert hut reading the same scriptural text over and over again. Jung’s protagonist is puzzled by this seemingly unfulfilling, repetitive activity until Ammonius explains to him the art of deep reading. In a still later chapter, The Red One and Ammonius appear together. They have been traveling in each other’s company in the interim, out of view of Jung’s narrator, and each looks a little worse for wear, having partially assimilated each other’s qualities and both blaming Jung’s protagonist for their predicament. Nonetheless, both have been transformed in the process, as has Jung’s own sense of self.

There are some noteworthy features of this form of dialogical engagement from the perspective of dialogical theory. First, Jung’s “I”, who serves as protagonist and provides narrative continuity to the text, is an actual participant in the dialogues; as Shamdasani [31] has pointed out, the “I” in the *Red Book* dialogues is not to be identified with Jung himself but is, itself, a full-fledged dialogical character in its own right. In contrast with Hermans’s dialogical self theory, where the “I” serves to give voice to various dialogical positions, in *The Red Book* dialogues the “I” is, itself, a dialogical position. Moreover, the characters in Jung’s narrative function autonomously with, as it were, their own voice, rather than needing to be voiced through the external agency of the “I.” Finally, as exemplified by the interaction between The Red One and Ammonius, these internal dialogues can take place entirely unconsciously, out of view of the “I”’s intentional control.

In the final part of *The Red Book*, the figure of Philemon finally emerges as the most authoritative voice amongst Jung’s interlocutors—the ultimate personification of Personality No. 2—and plays a critical role in fostering a sense of “otherness” within the self. Jung later wrote:

Philemon and other figures of my fantasies brought home to me the crucial insight that there are things in the psyche which I do not produce, but which produce themselves and have their own life. Philemon represented a force which was not myself. In my fantasies I held conversations with him, and he said things which I had not consciously thought. For I observed clearly that it was he who spoke not I.

([30], p. 183)

### 3.3. Dialogical Psychotherapy

In the subsequent development of his theory and practice, inner dialogue with an imaginary other went from being a merely personal preoccupation to an actual method of self-exploration and therapy. The dialogical approach that Jung developed in *The Red Book* was subsequently elaborated into the method of *active imagination*, which became the cornerstone of Jungian psychotherapy. In one of the few places in Jung’s *Collected Works* where he took up the theme of inner dialogue explicitly, he acknowledged that: “To anyone accustomed to proceed purely intellectually and rationally, this may seem altogether too ridiculous” ([32], para. 322); but he then went on to say that:

I mean this as an actual technique. We know that practically everyone has not only the peculiarity, but also the faculty, of holding a conversation with himself. Whenever we are in a predicament we ask ourselves (or whom else?), “What shall I do?” either aloud or beneath our breath, and we (or who else?) supply the answer. Since it is our intention to learn what we can about the foundations of our being, this little matter of living in a metaphor should not bother us.

([32], para. 323)

Jung also came to recognize dialogical engagement as essential to the *interpersonal* process of psychotherapy. In a late essay entitled “Principles of Practical Psychotherapy”, Jung described the therapeutic process as “a dialogue or discussion between two persons”, where: “A person is a psychic system which, when it affects another person, enters into reciprocal reaction with another psychic system” ([33], para. 1). This confrontation with an unknown other fosters unpredictable and uncontrollable outcomes that ultimately require the therapist to give up any pretentions to expert knowledge or authority. A genuinely dialogical relationship with another must afford full voice and authority to each participant, notwithstanding the constraints inherent in the asymmetrical roles of “therapist” and “client”. Dialogical psychotherapy, as Jung understood it, stood in stark contrast to proceduralized forms of therapy (what today is called “manualized” therapy), where psychotherapy is understood as “a method which anybody could apply in stereotyped fashion in order to reach the desired result” ([33], para. 1). Jung’s dialogical approach, in contrast, entails that the therapist let go of claims to expert authority and open up to the individuality of another in a way that changes the therapist along with the client. 

As Jung readily acknowledged, the dialogical approach is not meant for everyone. It is pointless, he suggested, “to subject a simple soul who lacks nothing but a dose of common sense to a complicated analysis of his impulses, much less expose him to the bewildering subtleties of psychological dialectic” ([33], para. 11); on the other hand, “with complex and highly intelligent people we shall get nowhere by employing well-intentioned advice, suggestions, and other efforts to convert them to some kind of system” ([33], para. 11). The second type of person, Jung argued, can best be helped by providing them an opportunity in a genuinely dialogical situation to develop and express their own uniquely individual understandings of their difficulties. It is the individuality of the person that is of paramount importance in dialogical psychotherapy, for “inasmuch as he is an individual he can only become what he always was” ([33], para. 11). This encapsulates the therapeutic objective of *individuation*, which became the hallmark of Jungian psychotherapy.

### 3.4. The Archetypal Background

Jung’s theoretical understanding of dialogical otherness within the self culminated in the notion of *archetype* and with the archetype of Self in particular. In his most definitive characterization of the notion, Jung identified the Self as the archetype that “expresses the unity of the personality as a whole” ([34], para. 789), which most often manifests as “a *complexio oppositorum*, a union of opposites” or a “united duality” ([34], para. 790). On this construal, the primary function of the Self is to unify contradictory tendencies of a person into an integrated whole. Here, as in his formulation of therapeutic method, dialogue merges with dialectic, consistent with Jung’s well-known penchant for dialectical thinking. Although Jung generally eschewed the “dry-as-dust philosophical dialectic” ([35], para. 286) and was no fan of the Hegelian formulation [36], his reference to the union of opposites and integrated wholes is plainly more dialectical than dialogical. Beebe [37] further developed a dialectical formulation of the archetypal Self by putting together Jung’s notion of archetypes with his theory of psychological types to produce an elaborate framework of opposites that is said to pattern dialogical activity within the Self. To the extent that these pairs of opposites are seen as something to be resolved, synthesized or integrated, however, the *centering* or *centripetal* tendencies of the Self are privileged at the expense of its non-integrative, *decentering* or *centrifugal* tendencies; in contrast, the dialogical self of Hermans and colleagues attempts to make room for both tendencies [8].

A more dialogical view of Jung’s archetypal Self opens up when it is seen as the archetype, not only of wholeness or unity, but of *otherness* itself. Papadopoulos referred to it as the “ultimate other” inasmuch as its alleged wholeness is always purely potential and thus lies forever beyond the bounds of what can be experienced. The archetypal Self is in this way ultimately and inescapably *other*; moreover, it is also, according to Papadopoulos, the “master archetype” or “archetype par excellence” ([27], p. 87). *Archetype* is a notion that Jung characterized in various ways in the *Collected Works*, both biologically and metaphysically. In his final substantive formulation of the theory of archetypes, Jung distinguished the *archetype as such* from its concrete expression in archetypal symbols. The *archetype as such*, wrote Jung, is fundamentally “irrepresentable”, inarticulate and unknowable; it constitutes “a background not previously suspected, a true matrix of all conscious phenomena” ([38], para. 356), a characterization to which he frequently returned in his subsequent work. Thus, archetypes in general, and the archetype of Self in particular, can be considered an aspect of background understanding, in the sense considered earlier in our discussion of the dialogical self.

However, while the Jungian archetypal psychology of Self and contemporary theories of the dialogical self thus share a notion of tacit, background understanding, they construe it differently. The dialogical background, as discussed earlier, is embedded in the *local background* of the immediate, communal contexts in which one is situated; whereas, the archetypal background is submerged in the *deep background* of the human way of being in the world that includes fundamental, existential concerns of human life [39,40,41]. This deep existential background constitutes a radical otherness that makes way for an unending depth of experience that is beyond “dialogue” in the ordinary sense; it is not so much a matter of relating to *an other* but, rather, to an indeterminate and undifferentiated *otherness* that constitutes the depths of unconscious life itself.

This difference, between the local and deep background, cuts to the heart of the tension between Jungian and postmodern construals of self. Zinkin has formulated the issue in terms of *essentialism versusconstructionism;* the issue, as raised in the title of his seminal essay, is: “Your Self: did you find it or did you make it?” [42]. Whereas Jung is traditionally understood as advocating the essentialist position of a pre-existing Self that subsequently structures experience, the constructionist position is that the self is something made rather than found; Zinkin, in particular, advocated the social constructionist view that “the self comes into experience only through interaction with others and the form it takes, the sense the individual has of being or having a self, will depend greatly on the culture in which he or she has been brought up” ([42], p. 394).

The issues at stake in Jungian *versus* postmodern approaches to the self are not so straightforward as the essentialist/constructionist dichotomy might suggest, however, given that, as Roesler [43] has pointed out, Jung continually fluctuated between both epistemological positions in his writings. Jones [44] advocated a dialogical “middle ground” position that recognizes the importance of both the embodied and social contexts of self, while acknowledging an ongoing tension between them. In dialogical terms, the local background of the socially constructed self constitutes what Papadopoulos [28] has called the “familiar other”, while the deep background of the archetypal self constitutes a “distant or exotic other”; and, as Papadopoulos went on to show, Jung tended to neglect the former and to unduly romanticize the latter. But the social constructionists have also tended to neglect the deep, archaic background of the embodied self, especially as manifest in non-conceptual and non-discursive modes of expression.

If the archetypal Self, as an aspect of deep background understanding, can never be fully articulated, then it cannot in the nature of things be reduced to a concept or hypothetical postulate in Jung’s system, much less to a dialectical synthesis of its existing postulates. As Huskinson has pointed out, “we do not have a substantial and precise theory of the Self because Jung did not develop one” ([45], p. 443). But what eludes conceptual understanding can nonetheless be enacted and expressed in non-literal, non-conceptual ways via visual art, myth, metaphor, religious ritual, and narrative fiction—in a word, *symbolically*. As Smythe and Baydala pointed out: “What cannot be captured adequately in concepts can nonetheless be hinted at, alluded to, or suggested through exemplifying, symbolizing, myth making, and storytelling” ([40], p. 66). For example, Jung found the dynamics of Self to be expressed in a diverse array of mythological motifs, including “the interplay of *yang* and *yin*, or of the hostile brothers, or of the hero and his adversary (arch-enemy, dragon), Faust and Mephistopheles, *etc.*” ([34], para. 790). Key to an understanding of such symbolic expressions of the archetypal Self is to distinguish the expressive function of symbols from the descriptive and explanatory functions of conceptual language. Smythe and Baydala [40], following Goodman [46], characterized symbolic expression as a form of metaphorical exemplification, that is, as a matter of metaphorically showing or presenting something, rather than literally defining or describing it. Symbolic expression thus opens up possibilities for the dialogical self beyond the domain of discursive and conceptual practices, to realms of the non-discursive and non-conceptual. There are substantial traditions of scholarship in philosophy, aesthetics and comparative religion on symbolic expression and the symbolic, non-conceptual uses of language that are potentially relevant here but which are beyond the scope the present paper to discuss at length.

## 4. Conclusions

The dialogical currents in Jung’s analytical psychology can both inform and be informed by contemporary developments in dialogical self theory. On the one hand, analytical psychology offers in some respects a more thoroughly dialogical perspective than dialogical self theory, inasmuch as the “I” or ego functions, itself, as a dialogical position amongst others within the self; these others are seen to function autonomously and with their own voice, beyond the intentional control and oversight of an “I”. Moreover, “dialogues” within the self can take place wholly unconsciously and non-discursively through non-conceptual modes of expression that reflect the deep archetypal background of embodied life, which opens the way to an undifferentiated *otherness* that goes beyond relationship to a specific *other*. On the other hand, dialogical self theory opens up dimensions of dialogical otherness hitherto occluded by analytical psychology. These include the decentering or centrifugal aspects of self that become evident when dialogue is detached from dialectic—the recalcitrant and “unmerged” voices that remain resistant to any form of dialectical synthesis or integration. Moreover, dialogical self theory highlights the local background of socioculturally embedded life and how it is taken up into the self, in a way that analytical psychology has traditionally been reluctant to do.

In neither the dialogical self nor the analytical psychology tradition, however, can the other within the self be considered an object of knowledge in the usual sense, viz., something that can be explicitly represented and subjected to truth claims. The background understanding of the other that is implicated in both traditions can only function as such so long as it remains tacit and hidden from view. When the “inner other” becomes foregrounded in experience as a participant in dialogue, it is invariably projected in an act of imagination that can never fully capture the unknown otherness that it expresses. While these imaginative constructions do not constitute discursive knowledge in any obvious sense, they do function to express, perform and exhibit our ongoing self-understanding in endlessly creative ways. How to properly evaluate such imaginative products for psychological meaning remains an open question.
